# High-quality draft genome sequence data of six *Lactiplantibacillus plantarum* subsp. *argentoratensis* strains isolated from various Greek wheat sourdoughs

**DOI:** 10.1016/j.dib.2021.107172

**Published:** 2021-05-28

**Authors:** Maria K. Syrokou, Spiros Paramithiotis, Panagiotis N. Skandamis, Eleftherios H. Drosinos, Loulouda Bosnea, Marios Mataragas

**Affiliations:** a Laboratory of Food Quality Control and Hygiene, Department of Food Science and Human Nutrition, Agricultural University of Athens, 75 Iera Odos St., 11855 Athens, Greece; b Department of Dairy Research, Institute of Technology of Agricultural Products, Hellenic Agricultural Organization “DIMITRA”, 3 Ethnikis Antistaseos St., 45221 Ioannina, Greece

**Keywords:** Bioinformatics, Genomics, Fermentation, Lactic acid bacteria, Lactobacillus, Sourdough, Whole-Genome Sequencing

## Abstract

*Lactiplantibacillus plantarum* is a species found in a wide range of foods and other commodities. It can be used as starter or adjunct culture in fermented foods. Herein the annotated high-quality draft genome (scaffolds) of six *L. plantarum* subsp. *argentoratensis* strains (LQC 2320, LQC 2422, LQC 2441, LQC 2485, LQC 2516 and LQC 2520) isolated from various Greek wheat sourdoughs is presented. Raw sequence reads were quality checked, assembled into larger contiguous sequences and scaffolds were annotated. The total size of the genomes ranged from 3.13 Mb to 3.49 Mb and the GC content from 45.02% to 45.13%. The total number of coding and non-coding genes were between 3268 and 3723 (3091 to 3492 protein-coding genes, 62 to 107 repeat-region, 54 to 59 tRNAs and 2 to 5 rRNAs, 20 to 30 crispr-repeats, 17 to 26 crispr-spacers and 2 to 4 crispr-arrays). The Whole Genome Shotgun project has been deposited at DDBJ/ENA/GenBank under the accession numbers JAEQMR000000000, JAEQMQ000000000, JAEQMP000000000, JAEQMO000000000, JAEQMN000000000 and JAEQMM000000000. The version described in this paper is version JAEQMR010000000, JAEQMQ010000000, JAEQMP010000000, JAEQMO010000000, JAEQMN010000000 and JAEQMM010000000. Raw sequence reads have been submitted in the Sequence Read Archive (SRA) under the BioProject accession number PRJNA689714 (BioSample accession numbers SAMN17215143, SAMN17215144, SAMN17215145, SAMN17215146, SAMN17215147 and SAMN17215148 and SRA accession numbers SRR13357463, SRR13357464, SRR13357465, SRR13357466, SRR13357467, SRR13357468).

## Specifications Table

**Table:** 

Subject	Food Science: Food Microbiology
Specific subject area	Genomics
Type of data	Table Figure
How data were acquired	Whole-Genome Sequencing: Illumina Novaseq 6000 (Illumina, CA) De novo assembly: Unicycler software as implemented in the PATRIC assembly web service (v3.6.8) Taxonomic assignment: Genome Taxonomy Database tool kit v1.1.0 (GTDB-Tk) as implemented in the KBase web service and KmerFinder v3.2 of the Center for Genomic Epidemiology (CGE) Server (http://www.genomicepidemiology.org/) Scaffolding: MeDuSa v1.6 web service Annotation: Rapid Annotation using Subsystem Technology tool kit (RASTtk) as implemented in the PATRIC annotation web service (v3.6.8). Annotation based on the NCBI Prokaryotic Genome Annotation Pipeline (PGAP) is also available at the NCBI website
Data format	Raw sequence reads, genome assembly and annotation
Parameters for data collection	Genomic DNA from pure microbial cultures
Description of data collection	Purification of genomic DNA, Whole-Genome Sequencing, genome assembly and genome annotation
Data source location	Institution: Laboratory of Food Quality Control and Hygiene of Agricultural University of Athens City/Town/Region: Traditional Greek wheat sourdoughs from various geographical regions Country: Greece
Data accessibility	*Raw data (reads)* Repository name: Sequence Read Archive (SRA) Data identification number: PRJNA689714 (BioProject) Direct URL to data: https://www.ncbi.nlm.nih.gov/sra/PRJNA689714 Repository name: Sequence Read Archive (SRA) Data identification number: SAMN17215143, SAMN17215144, SAMN17215145, SAMN17215146, SAMN17215147 and SAMN17215148 (BioSample) Direct URL to data: https://www.ncbi.nlm.nih.gov/sra/17215143, https://www.ncbi.nlm.nih.gov/sra/17215144, https://www.ncbi.nlm.nih.gov/sra/17215145, https://www.ncbi.nlm.nih.gov/sra/17215146, https://www.ncbi.nlm.nih.gov/sra/17215147, https://www.ncbi.nlm.nih.gov/sra/17215148
	Repository name: Sequence Read Archive (SRA) Data identification number: SRR13357463, SRR13357464, SRR13357465, SRR13357466, SRR13357467, SRR13357468 (SRA) *Assembled and annotated genomes* Repository name: DDBJ/ENA/GenBank Data identification number: JAEQMR000000000, JAEQMQ000000000, JAEQMP000000000, JAEQMO000000000, JAEQMN000000000 and JAEQMM000000000 Version number: JAEQMR010000000, JAEQMQ010000000, JAEQMP010000000, JAEQMO010000000, JAEQMN010000000 and JAEQMM010000000

## Value of the Data

• *L. plantarum* species is a microorganism found in a wide range of food commodities. Therefore, analysis of the genome of the *L. plantarum subsp. argentoratensis* strains will provide insights regarding their genomic and functional features and their potential use as a starter and/or adjunct culture
• Data could be of interest for third parties dealing with sourdough fermentations and/or other fermented foods, as well as with lactic acid bacteria as starters
• Data available to scientific community for applying other bioinformatics approaches such as comparative genomics to investigate the genome evolution of this species and other technological characteristics
• Contributing to the limited number of available genomes of the *L. plantarum* subsp. *argentoratensis* strain by providing high-quality whole-genome sequences

## 1 Data Description

**Fig. 1:**
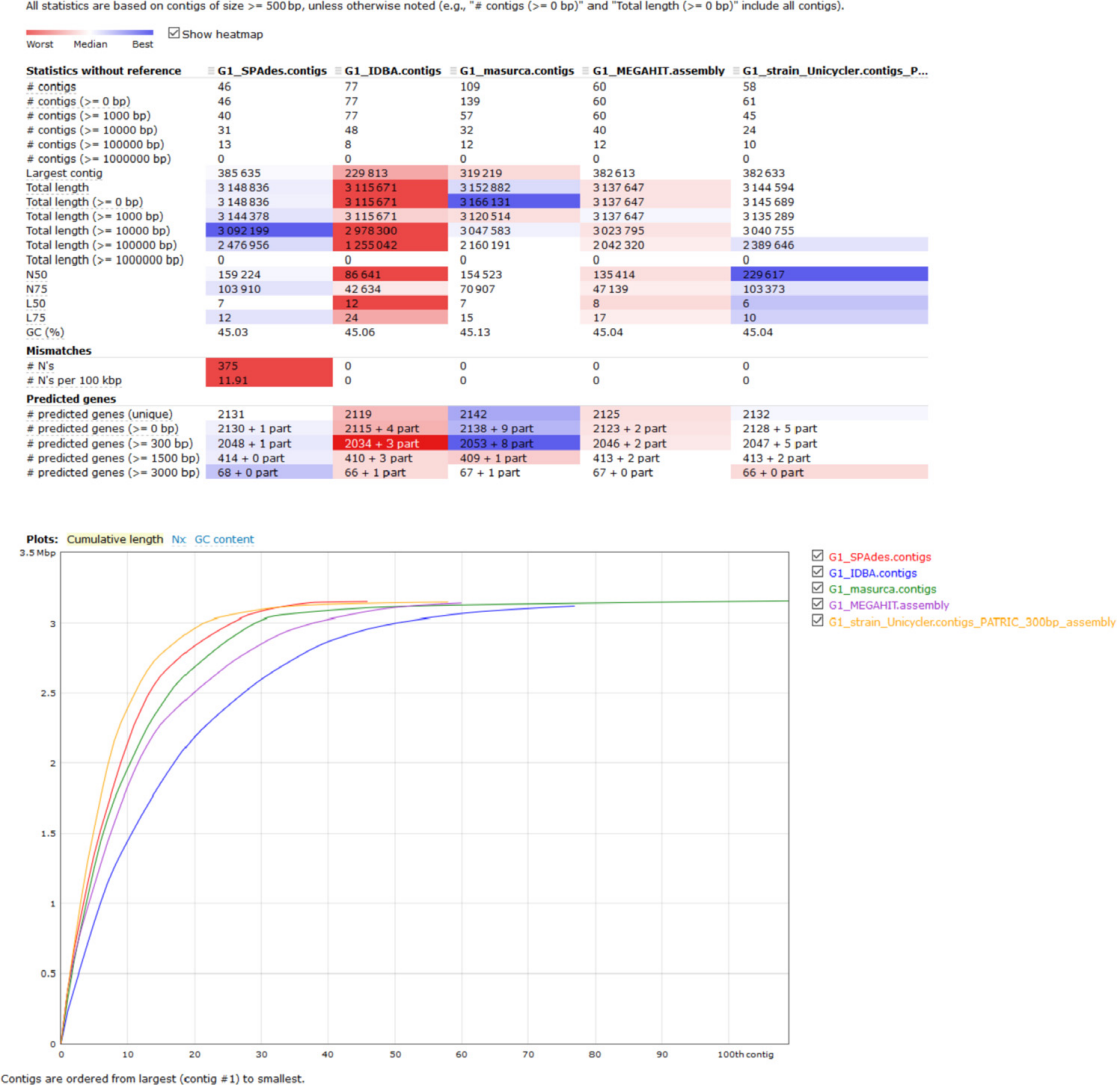
QUAST report comparing different assemblers for the *L. plantarum* subsp. *argentoratensis* LQC 2441 strain (assigned as G1_strain).

**Fig. 2:**
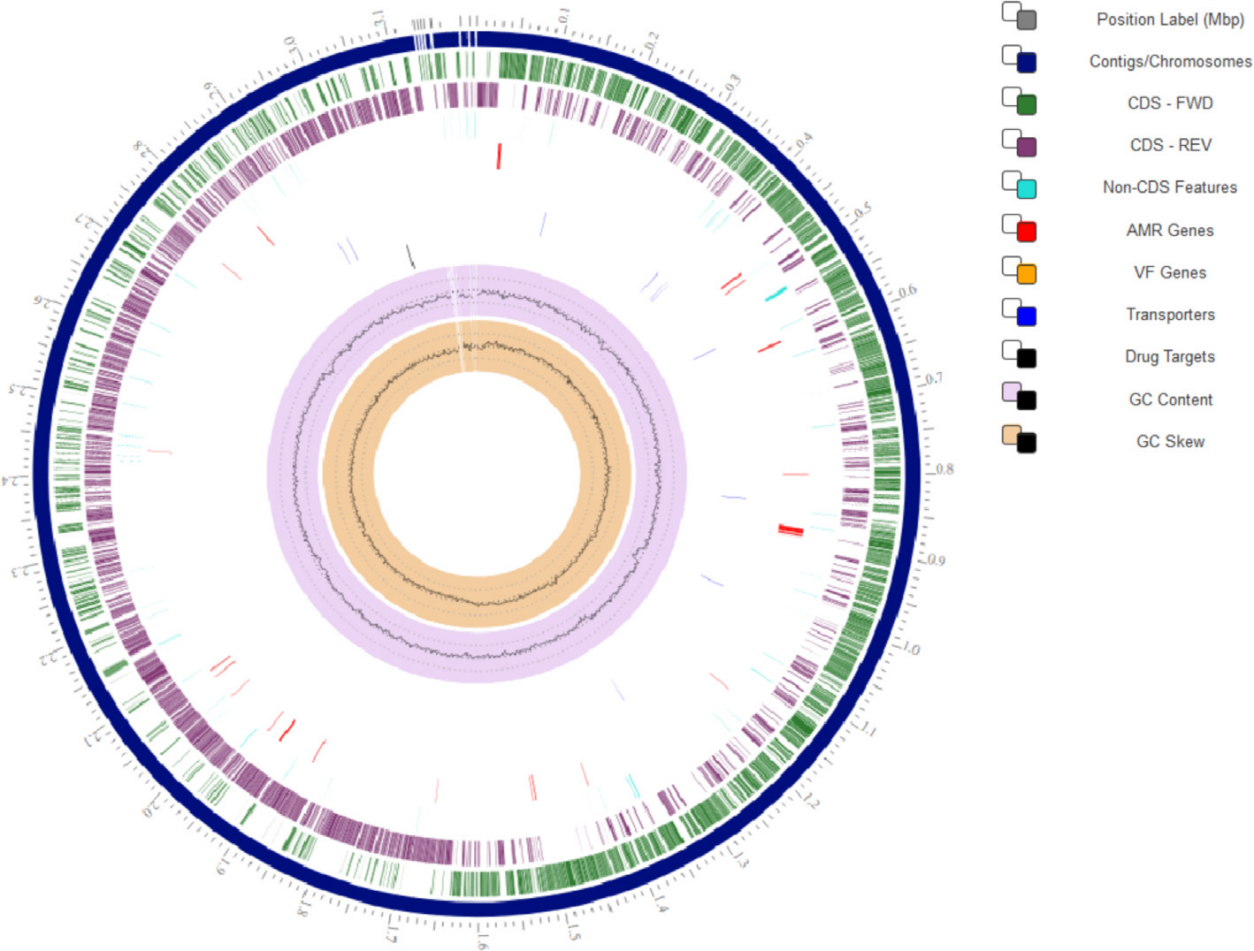
Circular view of the genome of *L. plantarum* subsp. *argentoratensis* LQC 2320 strain.

**Fig. 3:**
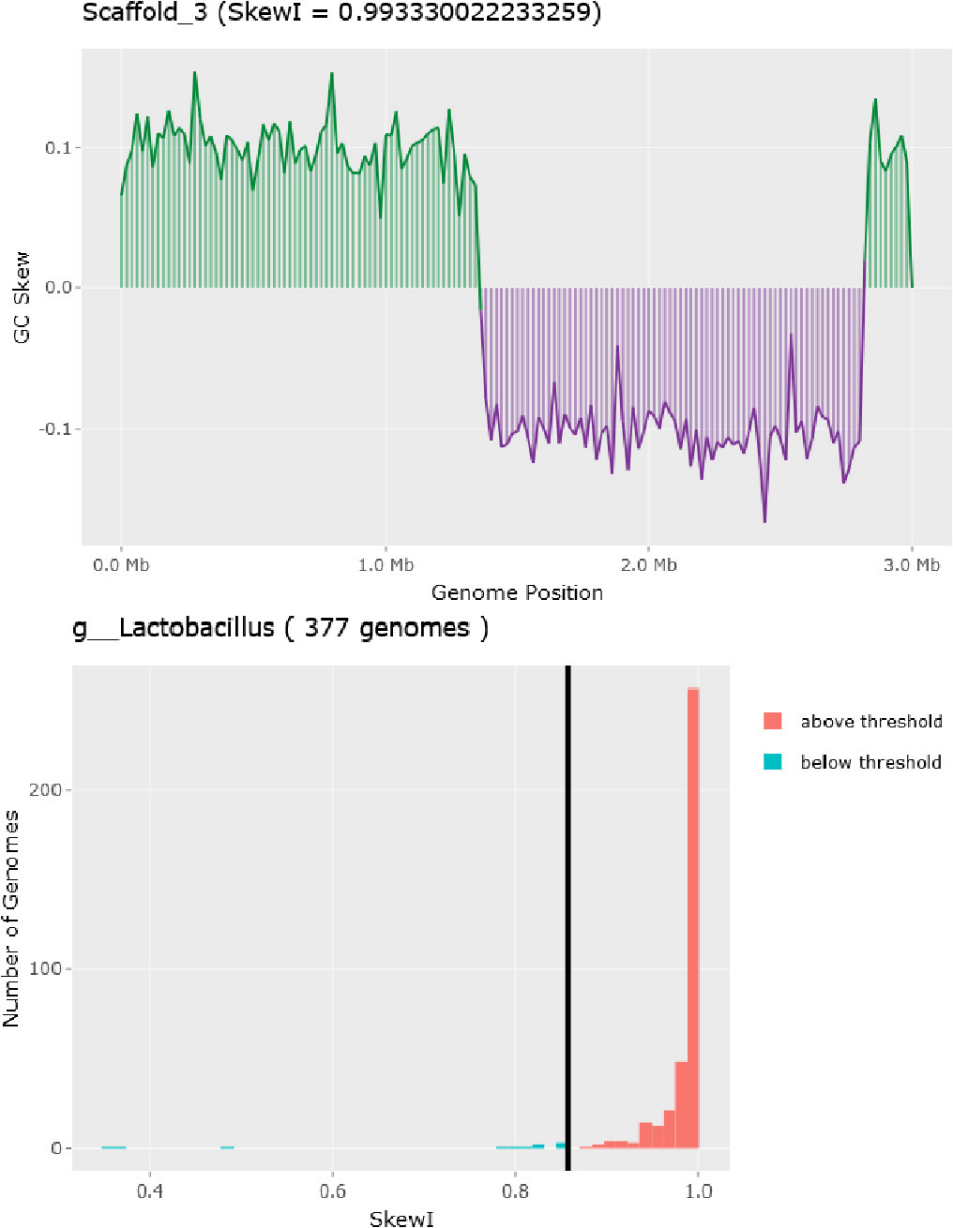
GC skew analysis of the genome of *L. plantarum* subsp. *argentoratensis* LQC 2516 strain (above) and skewI threshold value for the genus of *Lactobacillus* (below).

**Fig. 4:**
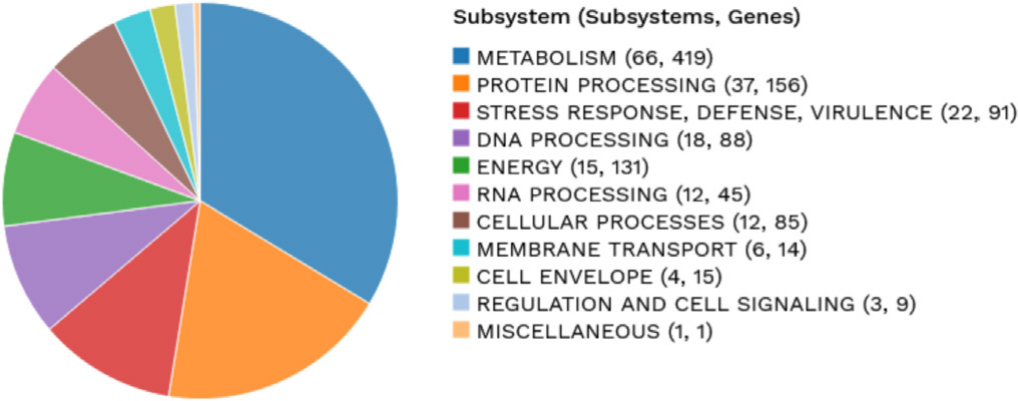
Subsystem analysis of the genome of *L. plantarum* subsp. *argentoratensis* LQC 2520 strain.

Herein the high-quality draft genome of six *L. plantarum* subsp. *argentoratensis* strains, isolated from Greek wheat sourdoughs [1], is presented. FastQC tool showed that the adapter-free raw reads were of high quality and therefore de novo assembly was performed without sequence trimming. Different assemblers were employed and QUAST revealed that in overall Unicycler provided the best assembly (Fig. 1). Quality metrics, genomic and functional characteristics of the genomes after scaffolding are shown in Tables 1 and 2, and Figure 2. CheckM, BUSCO and GC skew analysis confirmed the high quality of the genomes at scaffold level. Genome completeness (100%) and contamination (0% to 4.8%) levels were above and below the corresponding limits, respectively (>90% and <10%) (Table 1). Based on the BUSCO analysis, the percentage of BUSCO genes are displayed in Table 1 and the assembled scaffolds were free of contamination (i.e., the assembled sequences were screened against the NCBI UniVec database to quickly identify sequences of vector origin or those of adaptors or linkers). The SkewI metric ranged between 0.933 and 0.993 (Fig. 3; Table 1), which is far above the threshold value of 0.857 for the genus of *Lactobacillus* (Fig. 3). Quality of genome annotation was also good as represented by the genome annotation consistency indices and BUSCO evaluation (Table 2). The number of protein-coding genes annotated was 3091 to 3492 while the non-coding genes were between 160 and 231 (Table 2; Fig. 2). Subsystem analysis (set of proteins that perform a specific biological process or form a structural complex) depicted that almost 40% of the annotated protein-coding genes associated with metabolism followed by protein processing (*ca*. 15%) (Fig. 4). Finally, specialty genes related to transporters and antibiotic resistance were also identified (Table 2; Fig. 2).

**Table 1:** Characteristics and quality metrics of the six *L. plantarum* subsp. *argentoratensis* genomes after genome assembly into scaffolds^a^.

						CheckM		BUSCO			

Strain	No of scaffolds	Genome length (bp)	N50 (bp)	GC content (%)	SkewI metric	Completeness (%)	Contamination (%)	Complete and single copy (%)	Complete and duplicate copy (%)	Fragmented (%)	Missing (%)
LQC 2441	19	3,147,789	2,993,011	45.04	0.982	100	0.2	99.8	0.0	0.0	0.2
LQC 2485	46	3,494,755	3,148,808	45.02	0.934	100	4.8	99.3	0.5	0.0	0.2
LQC 2422	20	3,128,861	2,990,528	45.09	0.983	100	0.2	99.8	0.0	0.0	0.2
LQC 2320	8	3,181,752	3,129,011	45.13	0.991	100	0.0	100	0.0	0.0	0.0
LQC 2516	19	3,148,153	3,000,101	45.04	0.993	100	0.2	99.8	0.0	0.0	0.2
LQC 2520	10	3,175,498	3,140,405	45.12	0.987	100	0.0	100	0.0	0.0	0.0

a The percentage of Ns for each genome was 0.07% (LQC 2441), 0.12% (LQC 2485), 0.07% (LQC 2422), 0.05% (LQC 2320), 0.07% (LQC 2516) and 0.04% (LQC 2520).

**Table 2:** Quality and functional properties of the six *L. plantarum* subsp. *argentoratensis* genomes after genome annotation^a^.

		Non-coding						Consistency		BUSCO			
Strain	Protein-coding genes (CDS)	repeat-region	tRNA	rRNA	crispr-repeat	crispr-spacer	crispr-array	Coarse (%)	Fine (%)	Complete and single copy (%)	Complete and duplicate copy (%)	Fragmented (%)	Missing (%)
LQC 2441	3098	78	54	2	23	20	3	98.3	96.5	98.4	1.4	0.0	0.2
LQC 2485	3492	107	59	5	30	26	4	98.3	94.6	93.7	6.1	0.0	0.2
LQC 2422	3091	71	54	2	25	22	3	98.3	96.5	98.4	1.4	0.0	0.2
LQC 2320	3131	62	54	2	21	19	2	98.2	96.8	98.6	1.4	0.0	0.0
LQC 2516	3109	79	54	2	20	17	3	98.4	96.8	98.4	1.4	0.0	0.2
LQC 2520	3132	63	54	2	21	19	2	98.2	96.8	98.6	1.4	0.0	0.0

a Total number of genes for each genome was 3278 (LQC 2441), 3723 (LQC 2485), 3268 (LQC 2422), 3291 (LQC 2320), 3284 (LQC 2516) and 3293 (LQC 2520) of which the number of specialty genes was 38 (11 transporters and 27 antibiotic resistance for LQC 2441), 41 (11 transporters and 30 antibiotic resistance for LQC 2485), 38 (11 transporters and 27 antibiotic resistance for LQC 2422), 39 (12 transporters, 1 drug target and 26 antibiotic resistance for LQC 2320), 38 (11 transporters and 27 antibiotic resistance for LQC 2516) and 39 (12 transporters, 1 drug target and 26 antibiotic resistance for LQC 2520).

## 2 Experimental Design, Materials and Methods

*L. plantarum* subsp. *argentoratensis* strains were cultured in de Mann Rogosa and Sharpe (MRS) broth (LAB M, Lancashire, UK) and incubated overnight at 30°C. DNA was extracted from the microorganisms according to Syrokou et al. [1]. The genomic DNA was sequenced by Novogene Genomics Service (Novogene Co., Ltd, UK). At each step of the procedure (sample test, library preparation, and sequencing) quality control was performed. Agarose gel electrophoresis and Qubit 2.0 were employed to test DNA degradation and potential contamination, and to quantify the DNA concentration, respectively (sample quality control step). For the library construction and quality control, the genomic DNA was randomly fragmented by sonication, then DNA fragments were end polished, A-tailed, and ligated with the full-length adapters of Illumina sequencing, and followed by further PCR amplification with P5 and indexed P7 oligos. The PCR products as the final construction of the libraries were purified with AMPure XP system (Beckman Coulter, IN, USA). Then libraries were checked for size distribution by Agilent 2100 Bioanalyzer (Agilent Technologies, CA, USA), and quantified by real-time PCR. The qualified libraries were sequenced using paired-end (2 × 150 bp) libraries in the Illumina Novaseq 6000 sequencer (Illumina, CA, USA). Before assembling, adapter-free raw reads were quality checked with the FastQC v0.11.5 [2] tool of the KBase web service [3]. Different de novo assemblers such as SPAdes v3.13.0 [4], MEGAHIT v1.2.9 [5], IDBA-UD v1.1.3 [6] and MaSuRCA v3.2.9 [7], as implemented in the KBase web service, as well as Unicycler [8], as implemented in the PATRIC v3.6.8 assembly web service [9], were compared and the best assembler according to the Quality Assessment Tool (QUAST) v4.4 [10] (KBase) was selected to assemble reads into contigs. Pilon tool [11] accessible in PATRIC v3.6.8 assembly web service was used for polishing bacterial assembly. Taxonomic assignment of the assemblies was done through the Genome Taxonomy Database tool kit v1.1.0 (GTDB-Tk) [12] of the KBase and KmerFinder v3.2 [13] of the CGE Server (http://www.genomicepidemiology.org/). Contigs were organized into scaffolds using the Multi-Draft based Scaffolder (MeDuSa) v1.6 web server [14]. The scaffolds were ordered and orientated based on the complete genomes of *L. plantarum* subsp. *argentoratensis* DSM 16365 (GCA_003641165.1, ASM364116v1) and *L. plantarum* WCFS1 (GCA_000203855.3, ASM20385v3) used as reference genomes. A re-implementation of the algorithm of CheckM tool [15], offered by PATRIC v3.6.8, and BUSCO v3 [16] analysis with lactobacillales_odb9 dataset, facilitated through the GenomeQC web service [17], were employed to assess the genome quality at contig and scaffold level. In addition, potential bacterial mis-assemblies, after scaffolding, were evaluated with the Skew Index Test (SkewIT) web app [18]. Genome annotation of the scaffolds was performed using the Rapid Annotation using Subsystem Technology tool kit (RASTtk) [19] as implemented in the PATRIC v3.6.8 annotation web service. Quality of the genome annotation was assessed through the quality metrics provided by PATRIC annotation web service as well as through GenomeQC web service (BUSCO v3 with lactobacillales_odb9 dataset). Annotation based on the NCBI Prokaryotic Genome Annotation Pipeline, performed during the genome submission in the GenBank, is also available at the NCBI website (https://www.ncbi.nlm.nih.gov/).

## CRediT Author Statement

**Maria K. Syrokou:** Conceptualization, Investigation, Writing – review & editing; **Spiros Paramithiotis:** Conceptualization, Investigation, Supervision, Writing – review & editing; **Panagiotis N. Skandamis:** Supervision, Writing – review & editing; **Eleftherios H. Drosinos:** Supervision, Writing – review & editing; **Loulouda Bosnea:** Supervision, Writing – review & editing; **Marios Mataragas:** Conceptualization, Data curation, Formal analysis, Funding acquisition, Methodology, Project administration, Resources, Software, Supervision, Writing – original draft.

## Declaration of Competing Interest

The authors declare that they have no known competing financial interests or personal relationships which have or could be perceived to have influenced the work reported in this article.

## Data Availability

BioProject PRJNA689714 (Original data) (SRA/DDBJ/ENA/GenBank)
